# The Impact of Steroid Responder Status on Long-Term Outcomes in Critically Ill Patients With Acute Respiratory Distress Syndrome Receiving High-Dose Glucocorticoids

**DOI:** 10.7759/cureus.57445

**Published:** 2024-04-02

**Authors:** Thomas Leahy, Aneesha Chauhan, Victoria Nicholas, Pooja Patel, Alfred Wright, Samuel Miller, Geoff Ball, Christopher Remmington, Suveer Singh

**Affiliations:** 1 Intensive Care Unit, Royal Brompton Hospital, London, GBR; 2 Faculty of Medicine, Royal Brompton Hospital, London, GBR; 3 Anaesthesia, Southend University Hospital, Southend-on-Sea, GBR; 4 Research, University of Warwick, Warwick, GBR

**Keywords:** mortality predictors, extracorporeal membrane oxygenation support, invasive mechanical ventilation, lung injury score, response, pulmonary critical care, glucocorticoid therapy, systemic steroids, immunosuppression, acute respiratory distress syndrome [ards]

## Abstract

Background and objective

High-dose intravenous pulsed glucocorticosteroids (GCS) are not part of the standard treatment in acute respiratory distress syndrome (ARDS), and the evidence supporting their use is conflicting. In clinical practice, however, they are used in specialist settings when clinico-patho-radiological features suggest a potentially steroid-responsive pattern, or as a last resort in cases where patients are unable to be weaned off mechanical ventilation. This study aimed to investigate if an early objective response to high-dose GCS treatment in selected critically ill patients is predictive of survival in ARDS.

Methods

This study involved a case series of 63 patients treated at a tertiary specialist respiratory ICU between 2009 and 2017 who received high-dose GCS for ARDS following a multidisciplinary board agreement. Patients were stratified according to the change in their modified lung injury score (mLIS) between days 0 and 10 following GCS initiation. Changes in mLIS (range: 0-4) were grouped as follows - full responders: ≥2, partial responders: ≥1 and <2, and non-responders: <1. Mortality on discharge and at 6, 12, 18, and 24 months post-ICU discharge was assessed for each group. Data were analysed using logistic regression and a receiver operating curve (ROC) to determine a statistically significant association between the change in mLIS and survival.

Results

Of the 63 patients, there were seven full responders, 12 partial responders, and 44 non-responders to high-dose GCS. Overall mortality at ICU discharge and 6, 12, 18 and 24 months post-discharge was 29/63 (46.0%), 33/63 (52.4%), 34/63 (54.0%), 34/63 (54.0%), and 35/63 (55.6%) respectively. Mortality was significantly lower in the partial and full-response groups than in the non-response group at all time frames. Logistic regression showed a significant association between the change in mLIS and survival (p<0.001), and a ROC demonstrated that categorising the change in mLIS was a good predictive model for survival (c-statistic 0.86).

Conclusions

Measuring the change in mLIS by day 10 following high-dose GCS administration for ARDS may be clinically useful in prognosticating such patients. Further research using mLIS as a measure of response to GCS, and larger datasets to enable the evaluation of prognostic factors, may assist clinicians in predicting which patients with persistent ARDS are likely to respond to GCS therapy.

## Introduction

Despite the significant advances in ventilatory strategies for acute respiratory distress syndrome (ARDS), supportive care remains the cornerstone of its management [1]. In the absence of any specific pharmaceutical treatment for non-coronavirus disease 2019 (COVID-19)-related critically ill patients with ARDS, studies have analysed the use of glucocorticosteroids (GCS) as a disease-modifying agent on survival/mortality outcomes in ARDS. While some studies have demonstrated promising benefits with a class effect, such as a meta-analysis in 2015 [2], many of these have been small and single-centre in design with the potential for significant bias [1]. Indeed, several studies have found no difference or even worse outcomes associated with lower-dose GCS therapy, including an important randomised controlled trial (RCT) by the ARDS Clinical Trials Network, involving patients with ARDS persisting beyond one week [3,4].

These contradictory findings can be likely attributed to the heterogeneity of the study populations due to the broad range of aetiologies that can cause ARDS [1]. Furthermore, the timing of GCS intervention may also have an impact, depending on the degree and proportions of different pathophysiological processes (i.e., diffuse alveolar damage, organising pneumonia, and fibroproliferation) developing within the lung parenchyma during the clinical trajectory [5,6,7]. Even so, it is also possible that any potential short-term benefits of GCS are offset by concerns regarding the increased risk of infection, longer-term morbidity, and mortality associated with their adverse effects, in particular, their association with muscle wasting, and potential hindrance to the rehabilitation from ICU-acquired weakness. The impact of these factors has been challenging to quantify in trials [8]. Due to the risk of potential harm, and the lack of conclusive evidence of their benefit, higher-dose GCS therapy continues to be a non-standard treatment for ARDS [9].

Studies have also explored whether the use of GCS may be of benefit specifically in the inflammatory phase of ARDS (whereby patients exhibit possible ‘steroid responsive features’) [10] or in unresolving ARDS as a ‘rescue therapy’ [11]. In these contexts, higher-dose GCS therapy is used in specialist centres following clinico-patho-radiological consensus, despite mixed evidence regarding its benefits [12]. Selecting such patients remains a clinical, pathological, and radiological challenge for clinicians and no clear guidelines or biomarkers in isolation can identify patients with ARDS who respond to higher dose GCS [13].

Establishing whether there are true ‘responders’ and ‘non-responders’ to GCS and understanding the features that determine this is crucial for better-targeted therapy. An important step in this process involves validating robust tools that can standardise the assessment of clinical responsiveness to GCS in ARDS. Not only will this ensure potential responders receive therapy, but also spare non-responders from the adverse effects of GCS. In light of this, this case series aimed to identify if an early clinical and radiological response to high-dose GCS, using the established lung injury score (LIS) [14], is associated with improved survival in ARDS.

Our two primary hypotheses were as follows: (1) patients can be divided into distinct subsets depending on early clinical response (i.e., steroid-responsive and steroid-non-responsive patients); and (2) steroid-responsive patients have improved short- and medium-term survival outcomes. Conversely, the null hypothesis was that early clinical response is not associated with a significant difference in short- and medium-term survival.

## Materials and methods

Study design

Conventional LIS is calculated using chest X-ray (CXR) findings, the ratio of arterial oxygen concentration to the fraction of inspired oxygen (P/F ratio), positive end-expiratory pressure (PEEP), and compliance (tidal volume divided by driving pressure) (Table 1). Each parameter is scored, and an average is taken. This study used a modified LIS (mLIS) whereby the P/F ratio was removed for patients on extracorporeal membrane oxygenation (ECMO), and the score was calculated from the average of the remaining three parameters. The mLIS is the same as the conventional LIS for patients not supported with ECMO.

**Table 1:** The Murray lung injury score (LIS) for acute lung injury A matrix showing the criteria for calculating the Murray LIS [14]. The LIS is equal to the mean score from all domains, with higher scores indicating more severe lung injury. For the mLIS, the P/F ratio is omitted and the score is equal to the mean of the scores in each of the other domains CXR: chest X-ray; P/F ratio: the ratio of arterial oxygen concentration to the fraction of inspired oxygen; PEEP: positive end-expiratory pressure

Domain	Domain score				
	0	1	2	3	4
Consolidation on CXR	None	1 quadrant	2 quadrants	3 quadrants	4 quadrants
P/F ratio	>300 mmHg	225-299 mmHg	175-224 mmHg	100-174 mmHg	<100 mmHg
PEEP (cmH_2_O)	<5	6-8	9-11	12-14	>15
Compliance (ml/cmH_2_O)	>80	60-79	40-59	20-39	<19

To determine if there were separate subsets of responsive and non-responsive patients, patients were grouped as follows according to the reduction in mLIS scores between day 0 and day 10 following high-dose GCS administration: >2 for full responders, <2 but >1 for partial responders, and <1 for non-responders. These cut-offs were selected as each additional point on the LIS has been shown to be associated with a significant increase in morbidity and mortality in ARDS [13]. To test the null hypothesis, mortality was compared between these groups, and a logistic regression was performed to establish whether a relationship exists between early clinical response and mortality.

Inclusion criteria

A previous retrospective case-matched study [15] identified 78 patients at a tertiary specialist respiratory ICU between 2009 and 2017 who received high-dose GCS for ARDS diagnosed according to the Berlin criteria (Table 2) [16]. High-dose was defined as >500 mg of methylprednisolone intravenously within a 10-day period. We selected all patients in the intervention arm of the previous study who received at least one dose of >500 mg of methylprednisolone.

**Table 2:** The Berlin criteria* for ARDS *[16] ARDS: acute respiratory distress syndrome

Feature	Criteria
Onset	<7 days since onset of predisposing clinical condition
Chest imaging	Bilateral opacities – not fully explained by pleural effusion, collapse, or nodules
Cause of oedema	Respiratory failure not fully explained by cardiac failure or volume overload
Oxygenation [P/F ratio with >5 cmH_2_O of positive end-expiratory pressure (PEEP)]	Mild: 201-300 mmHg, moderate: 101-200 mmHg, and severe: < 100 mmHg

Exclusion criteria

Patients were excluded if it was not possible to calculate their mLIS; for instance, in cases where patients were not ventilated during their admission or had inadequate record keeping. Patients with a history of interstitial lung disease (ILD) were also excluded.

Data collection

mLIS scores were calculated on day 0 and day 10 following GCS administration. In cases where patients were extubated, discharged, or had died by day 10, the latest available data were used.

Data were collected retrospectively using electronic healthcare records. CXRs were scored according to the Berlin criteria, by the number of quadrants with consolidation. This was performed by two separate assessors and, in cases of conflicting scores, a third (the senior author) arbitrated. A score was agreed upon only once a consensus was reached by two assessors. This procedure was blinded, as X-rays were scored before calculating the rest of the mLIS and patient responder stratification. P/F ratios were calculated by dividing the PaO_2_ on the first arterial blood gas on day 0 and day 10 by the corresponding FiO_2_ at those times. Compliance and PEEP were calculated from the tidal volumes and pressures recorded at midnight on the relevant days. By standardising the timings used for data collection, we aimed to minimise potential information bias. Mortality at discharge and at 6, 12, 18, and 24 months post-discharge was collected for each group for comparison.

Statistical analysis

Descriptive data were expressed as mean (± SD) for continuous variables and proportion (%) for categorical variables. CXR, PEEP, P/F ratio, compliance scores, and mLIS at days 0 and 10 were compared among subjects by using Wilcoxon signed-rank tests. P-values and Rank-Biserial Correlations (RRb) were quoted. Potential contingency between ECMO use and responder status was assessed using Chi-squared analysis.

We tested the statistical significance of the change in MLIS score by using logistic regression. Logistic regression is used for binary dependent variables, as it can model the non-linear relationship between the independent variable and the probability of the dependent variable. This uses the logistic function:

Pr(Death|(ΔMLIS score) = 1/(1 + e­­-(α + βΔMLIS score))

The null hypothesis was that the change in MLIS score is not a significant indicator of the probability of death:

Ho: β = 0

The alternative hypothesis was that the change in MLIS score is a significant predictor of the probability of death, i.e., a fall in the MLIS score means that the patient is less likely to die.

H1: α > 0

A c-statistic was also calculated to evaluate the strength of responder status in predicting mortality. All analyses were performed using Python (Python Software Foundation, available at www.python.org) and Microsoft Excel.

## Results

**Figure 1:**
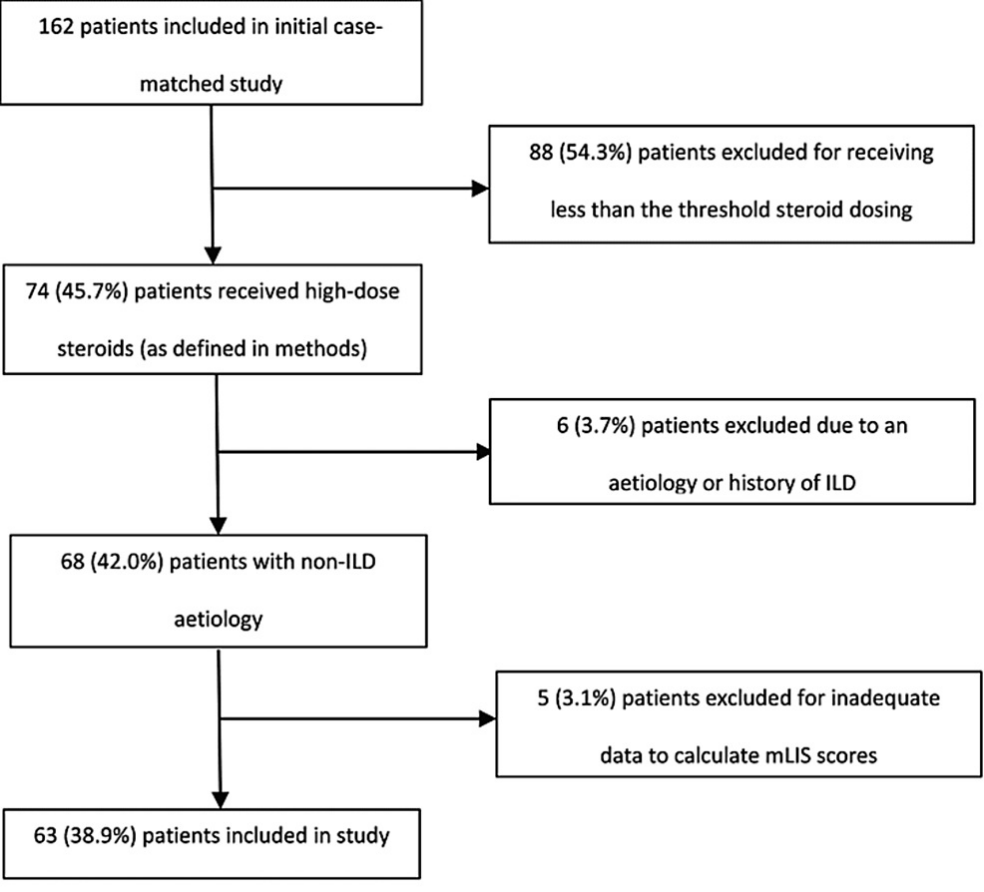
Consort diagram depicting the inclusion and exclusion of patients

From the 162 patients included in the previous case-matched study, this case series selected 63 patients from the intervention cohort of 74, who met the inclusion criteria for analysis. This represents approximately 10% of patients admitted to this ICU with ARDS. The reasons for exclusion are displayed in Figure 1. The average age on admission among those included was 51.5 years (±15.2); 37/63 (58.6%) of patients were male and the mean BMI was 27.8 kg/m^2^ (±6.3). The mean dose of methylprednisolone-equivalent GCS given over the 10-day sampling period was 2,881 mg (­±1,021).

**Figure 2:**
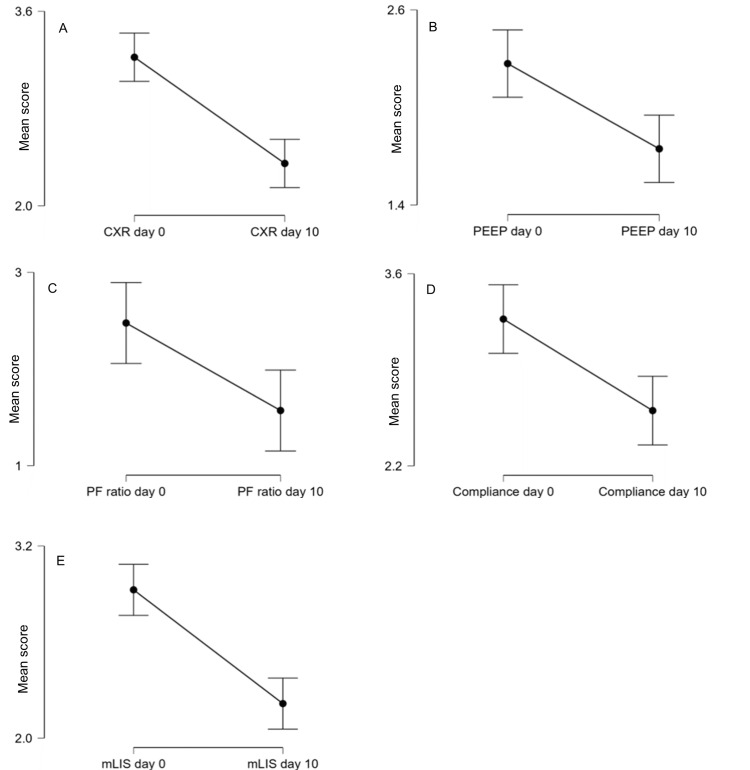
Average mLIS scores: overall and by parameter on days 0 and 10 This figure displays the mean score and 95% confidence intervals for each domain of the Murray LIS as well as the overall mLIS on day 0 and day 10 following high-dose GCS therapy. The scores of each LIS domain are displayed for days 0 and 10. All of these were lower on day 10 than on day 0; A - CXR score (p<0.001, RRb = 1), B - PEEP (0.001, RRb = 0.561), C - P/F ratio (p = 0.008, RRb = 0.775), D - compliance (p<0.001, RRb = 0.638) and E - overall mLIS (p<0.001, RRb = 0.848). Note that the P/F ratio (C) (n = 21) does not include patients on ECMO GCS: glucocorticosteroids; CXR: chest X-ray; P/F ratio: the ratio of arterial oxygen concentration to the fraction of inspired oxygen; RRb: Rank-Biserial Correlations; PEEP: positive end-expiratory pressure; ECMO: extracorporeal membrane oxygenation

As shown in Figure 2, the mean mLIS was lower on day 10 following the initiation of glucocorticoids compared to day 0 (p<0.001). This was reflected across all mLIS parameters. Responder status was not statistically dependent on ECMO status (x^2^ = 0.672, p = 0.715) (see Appendices).

**Figure 3:**
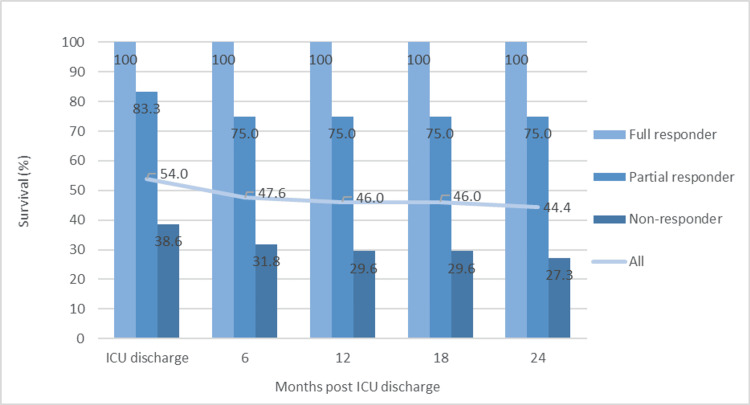
Survival following high-dose GCS therapy over time for each patient response group and the overall patient cohort This graph shows survival at various intervals (ICU discharge and 6, 12, 18, and 24 months post-ICU discharge) for each of the different responder groups as well as the entire cohort (see Appendices) GCS: glucocorticosteroids

Among the 63 patients treated with high-dose GCS, we identified 44 non-responders (69.8%), 12 partial responders (19.0%), and seven responders (11.1%). The survival at discharge and 6, 12, 18, and 24 months post-discharge for each response group is displayed in Figure 3. Mortality was highest among non-responders, followed by partial responders, and lowest for full responders.

**Figure 4:**
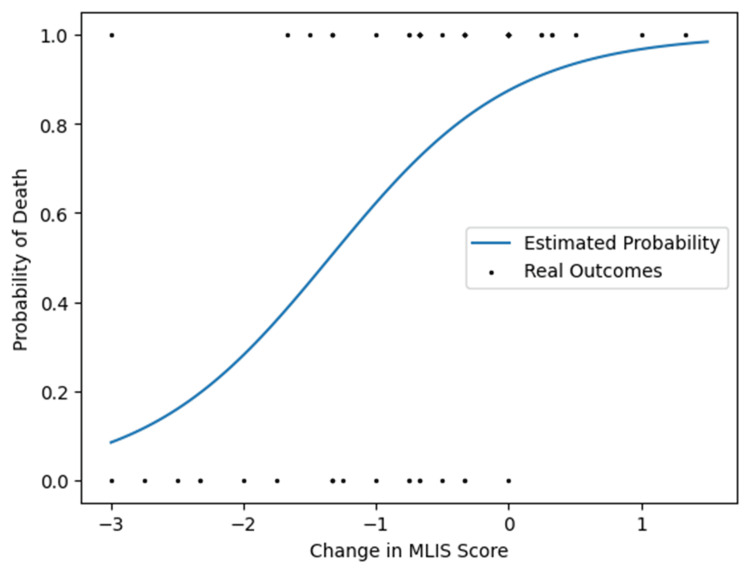
The effect of change in mLIS score between day 0 and day 10 on the probability of death A graph that shows the estimated impact of change in mLIS on the probability of death according to the logistic regression. Real outcomes are displayed by dots for each case, with 0.0 on the Y axis representing survival and 1.0 representing death at 24 months plotted against their measured change in mLIS score

The results from the logistic regression are summarised in Table 3. They show that the change in mLIS score was strongly significant (β > 0), with a p-value <0.001; hence, we reject the null hypothesis. There was significant evidence the change in mLIS score can predict the probability of death. A graphical representation of this data is shown in Figure 4.

**Table 3:** Logistic regression A table showing the results of a logistic regression modelling the relationship between the change in mLIS and the probability of death according to the formula Pr(Death|(ΔMLIS score) = 1/(1 + e­­-(α + βΔMLIS score)). As seen, the probability of death is lowest for patients with the greatest reduction in their mLIS between day 0 and day 10, and highest for those with an increase or minimal reduction in their mLIS

	Coefficient	Standard error	P-value	95% CI
α	1.94	0.48	0.000057	1.00, 2.89
β	1.44	0.43	0.000829	0.60, 2.28

**Figure 5:**
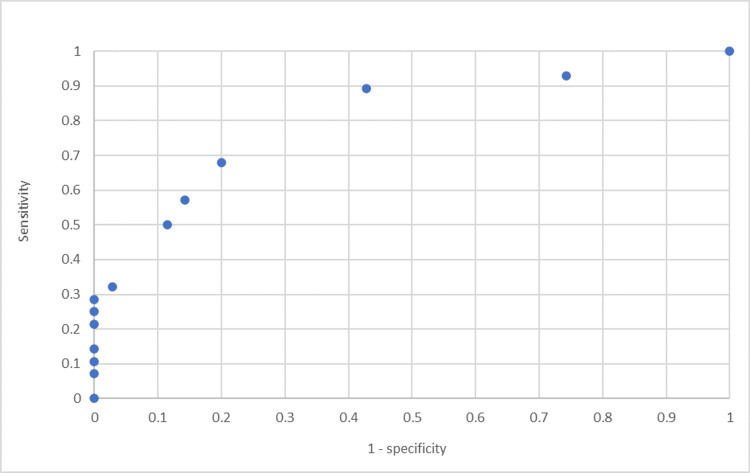
ROC curve for change in mLIS as a predictor for mortality at 24 months This ROC curve shows the true positive rate against the false positive rate for different thresholds of change in mLIS between day 0 and day 10 as a predictor for survival at 24 months. Predictive tools with potentially high sensitivity and specificity will have high y-axis values for low x-axis values. The c-statistic (area under the curve) calculated from this curve is 0.86 (range: 0-1, with higher values indicating a more reliable predictive tool)

A receiver operating characteristic curve was generated to illustrate the strength of the relationship between early response and mortality, as displayed in Figure 5. The c-statistic calculated from this curve is 0.86.

## Discussion

Several scoring systems exist for quantifying lung injury in ARDS. Higher LIS is associated with increased ARDS morbidity and mortality in ICU; however, studies have not demonstrated any superiority to the Berlin criteria for predicting outcomes [17]. The LIS was chosen in this case due to its multi-factorial inputs, and its ability for serial measurements, as opposed to the Berlin criteria's reliance on the P/F ratio. This was advantageous in this case series due to the presence of ECMO patients in the study for whom this parameter is invalidated. mLIS was used in this instance to allow for the scoring of patients on ECMO for whom the P/F ratio is not measurable reliably.

As no comparator control group was included in the study, we could not determine if high-dose GCS results in an overall improvement in survival. However, on average, patients experienced an improvement in their clinico-radiological status (measured by the delta mLIS scores) 10 days post-GCS initiation, as shown in Figure 2. Furthermore, logit regression modelling (Table 3 and Figure 4) showed that a reduction in mLIS was associated with increased survival (p<0.001). Patients who showed a partial or full steroid response by day 10 had lower mortality at all intervals (Figure 3). By inference, for these steroid-responsive patients, the clinical benefits of treatment outweigh the adverse effects. Additionally, mortality was concentrated in the immediate period following GCS administration with 29 deaths (46.0% mortality) at ICU discharge following high-dose GCS therapy, with a further six deaths (55.6% mortality) at 24 months. The quantification of responsiveness and identification of factors that predict steroid response becomes important for the effective targeting of such therapy in patients whose ARDS fails to improve. Cohort studies drawing on larger pooled databases are required to corroborate the indicators and implications from this case series.

Based on our finding that a reduction in mLIS score between days 0 and 10 is associated with lower mortality, we suggest that this may be a useful metric for measuring steroid response and prognosticating patients. The effectiveness of using the change in mLIS score in this context is represented by the ROC curve in Figure 5. The c-statistic of 0.86 demonstrates that this is a strong model for predicting survival at 24 months. In practice, this may aid clinical decision-making, particularly in the context of whether to continue active treatment for patients with ARDS during prolonged ICU stays. Furthermore, more accurate prognostication may be beneficial for patients and their relatives in providing information in an otherwise potentially uncertain clinical context. Finally, as there is such a strong link between change in mLIS score and survival, we propose that mLIS scores could be used as an outcome measure in future studies related to the use of high-dose GCS in ARDS to provide earlier results, thereby avoiding the need for follow-ups over several years.

Limitations

Due to the use of the mLIS, patients were not scored using common criteria depending on whether they were being supported by ECMO. It was considered that removing the P/F ratio would increase the variance in mLIS scores for ECMO patients and may disproportionately inflate or reduce the delta mLIS scores in this group. There was no statistical difference in the average change in mLIS scores between patients on ECMO (0.71) versus those not on ECMO (0.70) (x2 = 0.672, p = 0.715) (see Appendices). The parallels seen in the data for the two groups constitute reasonable evidence that the mLIS score is of similar value in both ECMO and non-ECMO patient groups.

A further limitation of the study involves the consideration of the impact of confounding factors. Several factors may independently affect both a patient’s GCS responsiveness and mortality. Furthermore, the impact of intercurrent infection, other factors that may cause a worsening of the CXR, and how they might modify the final LIS were not factored in. It would be beneficial if similar studies in the future could perform regression analyses to make up for these oversights; however, this would require a larger sample size than we included in this case series. Although it was not possible to account for all of the above-mentioned factors, all patients included in the sample were treated based on multidisciplinary team decisions made at the weekly board review meeting at a tertiary specialist respiratory ICU. Therefore, We believe the sample is quite representative of real-life practice and the conclusions we established are pertinent to developing further guidance on the use of high-dose GCS.

Finally, we did not perform a control group analysis performed this study. It could be argued that the degree of change in the mLIS in the 10 days sampled and the subsequent survival in the cases described here could have been part of the natural variation in the disease course of ARDS and that the administration of GCS was unrelated to these outcomes. Measuring an analogous 10-day period in a control group to better describe the natural variation in mLIS over time was considered; however, it was deemed impossible to pick an analogous cohort of patients for whom a multidisciplinary team would determine high-dose steroids to be of therapeutic benefit and who then were not administered the said steroids at that moment in time. Nevertheless, this is an aspect that could be of merit to address in future research through careful propensity matching.

## Conclusions

Measuring the change in mLIS by day 10 following high-dose GCS therapy for ARDS may be useful in identifying clinical response and prognosis in ARDS patients. This may aid clinicians in guiding their ongoing medical management. Further research to identify the features that predispose ARDS patients to be steroid-responsive would help better understand how to target such therapies. Measuring the change in mLIS as proposed in this study may represent an outcome metric that would be relatively simple to measure and be an effective surrogate for long-term mortality.

## Appendices

**Table 4:** Distribution of responder status and mean change in mLIS by ECMO status A table showing the relative proportion of non-, partial-, and full-responders and the change in mean mLIS for patients who did or did not receive ECMO. Responder status was not statistically dependent on ECMO status (x^2^ = 0.672, p = 0.715)

	ECMO (n = 42)	Non-ECMO (n = 21)
Non-responders (%)	69.0	71.4
Partial responders (%)	21.4	14.3
Full responders (%)	9.5	14.3
Change in mLIS	0.71	0.70

**Table 5:** Mortality at each interval by responder status A table showing cumulative mortality at ICU discharge and 6, 12, 18, and 24 months post-discharge for all cases and subsequently divided by responder status. This data is presented graphically in Figure 3

Response	Cumulative mortality over time (%)					24-month survival (%)
	ICU discharge	6 months	12 months	18 months	24 months	
Total sample (n = 63)	46.0	52.4	54.0	54.0	55.6	44.4
No response (n = 44)	61.4	68.2	70.5	70.5	72.7	27.3
Partial response (n = 12)	16.7	25.0	25.0	25.0	25.0	75.0
Full response (n = 7)	0.0	0.0	0.0	0.0	0.0	100.0
